# The nutritional status and root development of silver fir (*Abies alba* Mill.) seedlings growing on decaying deadwood in temperate forest ecosystem

**DOI:** 10.1038/s41598-023-45187-z

**Published:** 2023-10-19

**Authors:** Ewa Błońska, Jarosław Lasota, Marta Kempf, Ivika Ostonen

**Affiliations:** 1 Department of Ecology and Silviculture, Faculty of Forestry, University of Agriculture, Al. 29 Listopada 46, 31-425 Kraków, Poland; 2 Institute of Ecology and Earth Sciences, University of Tartu, Vanemuise 46, 51003 Tartu, Estonia

**Keywords:** Plant sciences, Ecology, Forest ecology, Forestry, Solid Earth sciences

## Abstract

The study aimed to compare two substrates, soil and deadwood, for the regeneration of silver fir (*Abies alba* Mill.) seedlings. Three-year-old fir seedlings growing both on deadwood and in the soil were collected. The examination involved determining the physical, chemical, and biochemical properties of soil and deadwood, as well as assessing the morphology of the roots and the nutrition of seedlings growing on the soil and deadwood. The examined substrates differed in physical, chemical and biochemical properties. It was shown that strongly decomposed fir logs are a good substrate for the growth of fir seedlings, mainly due to the high content of exchangeable cations (especially calcium, magnesium and potassium) and high phosphorus and nitrogen content. The type of substrate had a significant impact on the root morphology of fir seedlings. In our study, the most responsive root traits to differences in growing substrates were specific root area (SRA) and specific root length (SRL). Our analyses did not confirm significant differences in the stoichiometry of C, N and P in the roots and needles of seedlings grown on different substrates. The stoichiometry of roots and needles suggests no limitations in the uptake of nutrients by seedlings growing on deadwood. This study validated that heavily decomposed wood can provide favourable microhabitats for the growth of the young generation of fir.

## Introduction

Deadwood is an essential element of the forest ecosystem, which affects the formation of soil properties, both physical, chemical and biochemical^1–3^. It serves as a habitat for a myriad of plants and animals, including lichens, bryophytes, fungi, invertebrates, birds and mammals^4–7^. The importance of deadwood in fostering biodiversity within the understory has been well-documented in various studies^8–10^. Many plant species find favourable conditions for regeneration on decaying wood^5,6^. The properties of deadwood exert a notable impact on seedling establishment, growth, height distribution and overall survival^11^. Previously conducted studies indicate a relationship between the degree of decay of deadwood and the impact on the physical, chemical and biochemical properties of soils^12,13^. During the decomposition of deadwood, both deciduous and coniferous species, their density, humidity and porosity change, which has consequences for the amount of released components^12^. Seedlings, in particular, benefit from the improved humidity and thermal conditions offered by deadwood^14,15^. The study confirmed significant differences in the C, N and P stoichiometry of decaying wood depending on the degree of decomposition and the soil affected by the deadwood^13^.

Germination, survival and growth of seedlings depend on such environmental conditions as temperature, moisture and nutrients^15^. Seed viability, dispersal, germination and mycorrhizae are critical to the seedlings' establishment^16,17^. Additionally, potential substrates for seedling regeneration, such as the organic layer, mineral soil or deadwood, exhibit significant variations in their physical and chemical properties^18,19^. The study shows that regeneration processes are determined by soil moisture, organic C content and pH of the surface horizon^20,21^. The root system's status and functioning determine the seedlings' quality^22^. Roots are responsible for nutrient uptake, and their capacity to adapt to varying environmental conditions enables plants to expand their ecological niche^23,24^. These adaptations may manifest through morphological, physiological, or chemical traits^25^. The knowledge about morpho-physio-phenological traits of roots offers insights into how roots influence the functioning of an ecosystem^26^.

The study focused on silver fir (*Abies alba* Mill.), the primary forest-forming species in a temperate climate. Natural regeneration of fir is becoming increasingly important in forest management, and investigating the role of deadwood as a potential microhabitat for this species' growth can provide valuable insights. Understanding the forest recovery process and identifying the factors influencing silver fir seedling regeneration on the deadwood is significance. So far, no studies have been conducted that simultaneously analysed the nutrition of seedlings and the morphology of their root systems in relation to the properties of soil and deadwood. Deadwood as a substrate for the growth of seedlings is a relatively unexplored area compared to other substrates such as peat or biogas residues^27,28^. Our research fills this gap. The purpose of the research was: (1) to assess the physical, chemical and biochemical properties of deadwood in the advanced degree of decomposition in which a new generation of fir appears; (2) to compare the nutritional status of seedlings growing on deadwood at an advanced stage of decomposition and soil; (3) to compare the root morphology of seedlings growing on deadwood with seedlings growing in soil.

## Materials and methods

### Study site and experiment design

Our study was conducted in Czarna Rózga Reserve in Central Poland (50° 59′ N, 20° 01′ E). In the study area, the average annual rainfall is 649 mm, the average annual temperature is 7.4 °C, and the length of the growing season is 200–210 days^12^. The study plots were dominated by Gleysols, Cambisols and Podzols developed on fluvioglacial sand and loam. In the stands of the reserve covered by the study, there are common alder (*Alnus glutinosa*), silver fir (*Abies alba*), pedunculate oak (*Quercus robur*), common hornbeam (*Carpinus betulus*) and common ash (*Fraxinus excelsior*)^26^. The research was carried out in parts of the reserve with mixed forest stands (fir, hornbeam and alder). Thirty research plots were selected for study in a similar geographic location (flat terrain) and a similar tree stand (90 years old, moderate canopy density). We selected 15 study plots with fir logs with a diameter between 25 and 35 cm covered with fir regeneration. At the same time, 15 study plots with the regenerated fir seedlings growing on the Gleysols were selected for the analysis. Logs of silver fir at the V decay class were selected for the analysis. The decay class was determined using the Maser classification^2^. Wood in the V decay class is characterized by a soft and loose texture, oval shape, faded color of wood. Wood at this stage of decomposition has no bark and branches and entirely on the ground. The soil samples and wood samples from each study plot were collected. All wood and soil samples for analysis were taken in September 2018. The wood samples were collected from the same decaying logs on which the fir seedlings grew. Soil samples were taken from a depth 0–10 cm using a plastic spade. Fifteen wood samples and 15 soil samples were taken for further laboratory analysis. Three-year-old fir seedlings growing both on deadwood and in the soil were collected. Seedling age was determined by the presence of a lateral shoot, which in fir trees develops in the third year of life^27^. Ten seedlings were taken from each study plot. A total of 300 seedlings were taken for analysis.

### Laboratory analysis

After drying to an air-dry state and sieving through a sieve with a diameter of 2 mm, soil and wood samples were analysed in laboratory conditions. The pH of the soil and wood samples was determined by the potentiometric method in water and KCl. Carbon and nitrogen content was determined using the Leco analyser (Leco, St. Joseph, MI, USA). The content of Ca, Mg, Na and K, as well as the content of Cd, Cr, Cu, Ni, Pb, and Zn, were determined using an ICP apparatus (ICP-OES Thermo iCAP 6500 DUO, ThermoFisher Scientific, Cambridge UK). Determination of Cd, Cr, Cu, Ni, Pb, and Zn content was preceded by mineralisation. Nitric acid was used to mineralise wood, and a mixture of nitric and perchloric acid was used to mineralise soil samples. Based on the obtained results, sums of bases (BC) were calculated. In samples of wood and soil with natural moisture, the activity of dehydrogenases (DH) was determined according to the Casida procedure^28^ and the activity of β-glucosidase by the method of Eivazi and Tabatabai^29^. Microbial biomass carbon (MBC) and nitrogen (MBN) were determined in soil and wood samples using CHCl3 fumigation^30^. The amount of carbon and nitrogen in soil and wood samples was quantified^31^.

The micro and macroelement content in the needles and roots of the studied seedlings was determined using ICP after prior mineralisation in nitric and perchloric acid. Nitrogen and carbon content in needles and roots was determined using a Leco analyser. Based on the obtained results, the stoichiometry of C, N and P in needles and roots of seedlings was determined.

The entire root system of the seedlings was analysed, and the root systems were classified as fine roots with a diameter of less than 2 mm. Root morphology was assessed using the WinRhizo™ Pro 2003b image analysis system (Regent Instruments Inc., Ville de Québec, QC, Canada). Root images were acquired using a desktop optical scanner for subsequent analysis. Before analysing root morphology, the seedlings were carefully cleaned, and soil was removed from the root surface. After drying (70 °C, 48 h), both the root systems and the aboveground parts of the seedlings were weighed. Based on the obtained results, root tissue density (RTD), specific root area (SRA) and specific root length (SRL) were calculated^32^.

### Statistical analysis

Statistical analyses were conducted using Statistica 12 (StatSoft 2012). The normal distribution was assessed using the Shapiro–Wilk test. The U Mann–Whitney test determined differences in wood and soil properties, and the Pearson correlation was used to determine the relationship between the examined characteristics of wood and soil. Quantitative data were used in the Pearson correlation analysis, and a scatterplot was generated before conducting the analysis. In addition, principal component analysis (PCA) was employed to illustrate the relationship between the analysed properties.

### Statements

Our research and field studies on plants, including the collection of plant material, complied with relevant institutional and national legislation. We have the necessary permits to conduct research (Consent issued by the Regional Director for Environmental Protection in Łódź on January 23, 2017 (WPN-I.6205.78.2016.DB). Three-year-old fir seedlings, growing both on deadwood and in the soil, were the voucher specimen. Prof Jarosław Lasota conducted the identification. Voucher specimens had not been deposited in a public collection due to the use of all material in laboratory analysis.

## Results

### Properties of different substrates

**Figure 1:**
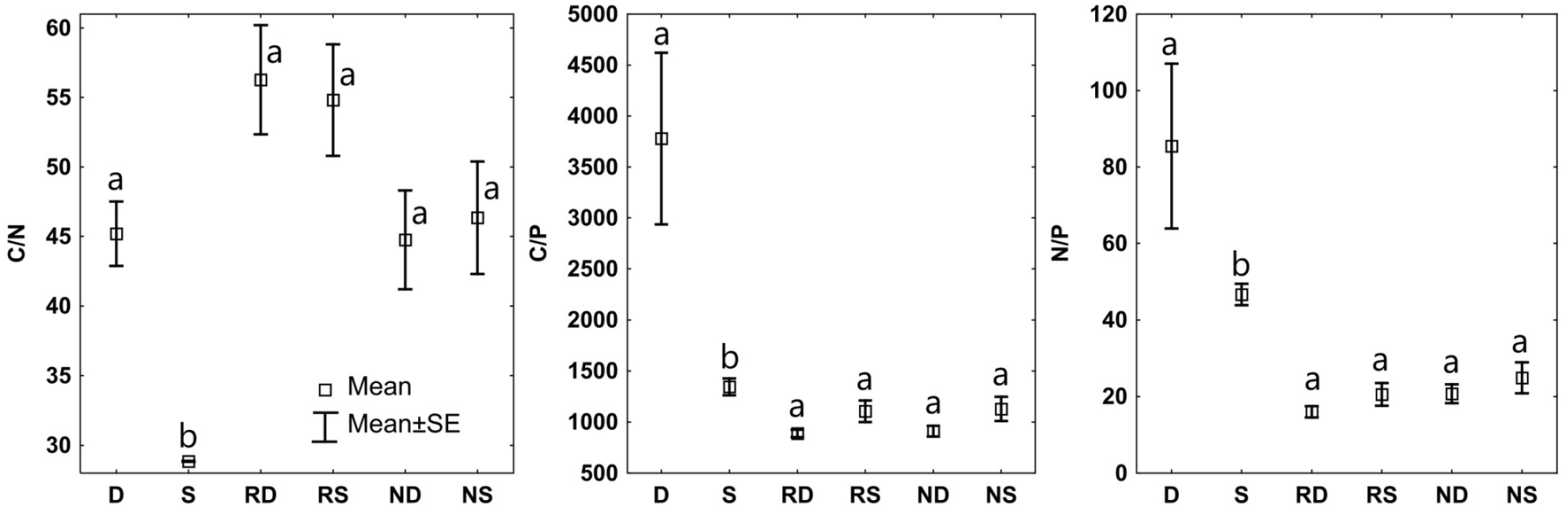
C:N:P stoichiometry of deadwood (D) and soil (S), needles (N) and roots (R) of seedling growing on soil and deadwood; small letters (a and b) mean significant differences between soil and deadwood.

The average carbon content in the soil was 14.90%, whereas in deadwood, it was notably higher at 40.92%. Significant differences in carbon content were noted between soil and deadwood samples (Table 1). Nitrogen content tended to be higher in deadwood, with an average of 0.86% in deadwood and 0.60% in soil. Statistically a significantly higher content of cations was noted in deadwood. BC of deadwood was 15.79 cmol ( +)^.^kg^−1^, and for soil it was 5.37 cmol ( +)^.^kg^−1^. No differences in pH or P content were noted. Differences in the substrates' C/N, C/P and N/P ratios were visible for the soil and deadwood. We recorded a higher C/N/P ratio in the deadwood samples than in soil samples, 3779:85:1 and 1345:47:1, respectively (Fig. 1). Statistically significant differences were noted in all examined physical properties (Table 1). Soil had higher bulk density compared to deadwood, while deadwood showed significantly higher moisture and porosity. Statistically, significantly higher (twofold) dehydrogenase and β-glucosidase activity were noted in deadwood (Table 2). The average dehydrogenase activity was 35.18 µmol TPF^.^kg^−1^ h^−1^ in deadwood and 11.37 µmol TPF^.^kg^−1^ h^−1^ in soil. The highest MBC and MBN were noted in deadwood samples.

**Table 1:** The chemical and physical properties of study soil and deadwood.

	C	N	C/N	pH H_2_O	BC	Ca	K	Mg	Na	P	BD	Mw	Mv	Por	Pa	CWC
Soil	14.90 ± 3.31^b^	0.60 ± 0.13^a^	24.73 ± 0.05^a^	3.77 ± 0.10^a^	5.37 ± 1.55^b^	3.91 ± 1.14^b^	0.52 ± 0.03^b^	0.85 ± 0.10^b^	0.08 ± 0.01^b^	283.30 ± 33.60^a^	0.71 ± 0.13^a^	21.10 ± 2.51^b^	14.83 ± 1.16^b^	71.00 ± 4.65^b^	18.17 ± 4.31^b^	52.87 ± 1.20^b^
Deadwood	40.92 ± 3.68^a^	0.86 ± 0.48^a^	63.18 ± 43.33^a^	3.99 ± 0.32^a^	15.79 ± 3.41^a^	12.04 ± 2.77^a^	0.95 ± 0.30^a^	2.62 ± 0.40^a^	0.16 ± 0.03^a^	322.52 ± 169.52^a^	0.13 ± 0.03^b^	70.17 ± 7.03^a^	47.90 ± 4.51^a^	89.80 ± 3.08^a^	30.40 ± 3.91^a^	59.40 ± 1.31^a^

Mean ± SD; soil samples n = 15, deadwood samples n = 15.

*C* carbon content (%), *N* nitrogen content (%), *BC* base cations content (cmol (+) kg^−1^), *P* available phosphorus (mg kg^−1^), *BD* bulk density (g cm^−3^), *Mw* weight moisture (%), *Mv* volumetric moisture (%), *Por* porosity (%), *Pa* air porosity (%), *CWC* capillary water capacity (%).

Different lowercase alphabets in the upper index mean significant differences of parameters.

**Table 2:** Biochemical properties of study soil and deadwood.

	DH	BG	MBC	MBN	MBC/MBN
Soil	11.37 ± 3.01^b^	7805.1 ± 151.5^b^	1772.3 ± 100.8^a^	292.4 ± 23.8^a^	6.07 ± 0.15^a^
Deadwood	35.18 ± 2.13^a^	13,274.7 ± 2603.1^a^	2821.3 ± 1338.1^a^	534.1 ± 273.2^a^	5.34 ± 0.39^b^

Mean ± SD; soil samples n = 15, deadwood samples n = 15.

*DH* dehydrogenase activity (µmol TPF^.^kg^−1^ h^−1^), *BG* β-glucosidase activity (mmol pNP kg^−1^ h^−1^), *MBC* microbial biomass carbon (µg g^−1^), *MBN* microbial biomass nitrogen (µg g^−1^).

Different lowercase alphabets in the upper index mean significant differences of parameters.

### Nutritional status of seedlings

We noted no significant differences in N, C and P content between the needles of seedlings growing on different substrates (Table 3). However, the substrate significantly influenced the C/N/P stoichiometric ratio in both needles and roots of the seedlings. In the needles of seedlings growing on deadwood, the stoichiometry of C and N relative to P was different compared to the corresponding stoichiometry in the needles of seedlings growing on soil (912/21/1 and 1129/25/1, respectively) (Fig. 1). We found a similar shift in the roots of the seedlings. In contrast, C/N/P stoichiometry was narrower in those growing on deadwood and broader in roots grown in the soil.

**Table 3:** Chemical parameters of seedling needles and roots growing on a different substrate.

	C (%)	N (%)	C/N (%)	P ( mg kg^−1^)	Ca ( mg kg^−1^)	Mg ( mg kg^−1^)	Na ( mg kg^−1^)	K ( mg kg^−1^)	Fe ( mg kg^−1^)	Cu ( mg kg^−1^)	Zn ( mg kg^−1^)	Mn ( mg kg^−1^)
Seedling needles												
Soil	47.14 ± 0.28^a^	1.20 ± 0.17^a^	39.74 ± 6.01^a^	1084.2 ± 163.3^a^	3732.7 ± 380.1^a^	1100.2 ± 28.03^a^	210.4 ± 43.9^a^	2552.3 ± 200.6^b^	123.7 ± 19.2^a^	26.0 ± 5.8^a^	45.1 ± 2.8^a^	236.9 ± 57.3^a^
Deadwood	47.72 ± 0.75^a^	1.26 ± 0.14^a^	38.37 ± 5.27^a^	1374.2 ± 123.8^a^	3475.3 ± 175.6^a^	867.4 ± 79.4^b^	171.0 ± 11.6^a^	3258.7 ± 182.2^a^	256.2 ± 106.0^a^	29.3 ± 3.8^a^	48.3 ± 9.6^a^	450.4 ± 178.8^a^
Seedling roots												
Soil	47.86 ± 1.06^a^	1.03 ± 0.11^a^	47.00 ± 5.96^a^	1138.3 ± 203.9^a^	2129.3 ± 405.0^a^	619.2 ± 19.2^a^	287.2 ± 114.3^a^	2397.7 ± 155.9^a^	693.0 ± 340.9^a^	32.8 ± 6.8^a^	76.2 ± 6.3^a^	113.4 32.4^a^
Deadwood	47.79 ± 0.53^a^	1.00 ± 0.11^a^	48.26 ± 5.83^a^	1389.5 ± 92.0^a^	2323.3 ± 165.2^a^	554.7 ± 52.6^a^	193.0 ± 21.0^a^	2689.3 ± 189.7^a^	219.2 ± 97.2^a^	29.4 ± 6.9^a^	81.4 ± 24.8^a^	175.9 ± 71.8^a^

Mean ± SD; seedlings growing on soil n = 150, seedlings growing on deadwood n = 150.

Different lowercase alphabets in the upper index mean significant differences of parameters.

### Morphological characteristics of seedlings

**Figure 2:**
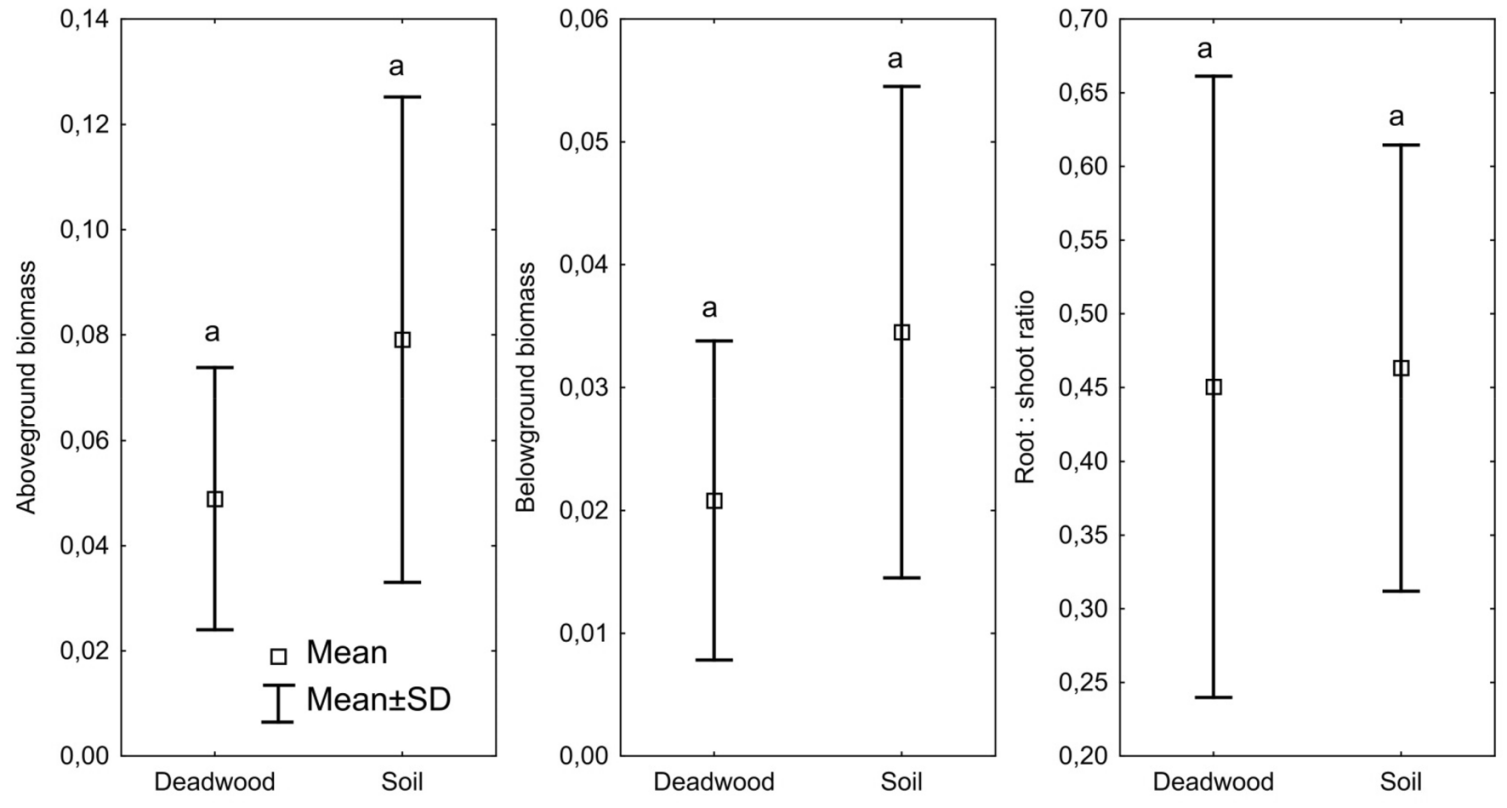
Aboveground biomass (g), belowground biomass (g) and root: shoot ratio of seedling growing on soil and deadwood; small letters (a and b) mean significant differences between soil and deadwood.

The seedlings growing on the soil had a higher average aboveground and belowground biomass, measuring 0.079 g and 0.034 g, respectively. In contrast, for seedlings growing on deadwood, the average aboveground biomass was 0.048 g, and the belowground biomass was 0.021 g (Fig. 2). There were no statistically significant differences in the ratio of root-to-shoot biomass between seedlings growing on deadwood and soil. The substrate variant had a significant impact on the root morphology of fir seedlings (Table 4). In the case of the roots of soil-grown seedlings, a higher length and weight were noted. The roots of seedlings growing on soil were statistically significantly longer. The specific root area was significantly higher in seedlings growing on deadwood. Also, specific root length tended to be higher in seedlings grown on deadwood (Table 4). Root tissue density did not differ between the studied variants. Significant correlations between root morphology and properties of different substrates were noted (Table 5). Positive correlations were found between SRA and C, Ca, and Mg content, as well as the activity of dehydrogenases and β-glucosidase. SRA also correlated positively with C/N, C/P and N/P ratios (r = 0.40, r = 0.49 and r = 0.48, respectively). A similar relationship was noted in the case of SRL and the C/N, C/P and N/P ratios (Table 5). Additionally, the conducted research confirmed the statistically significant relationship between the roots' morphological features and the tested substrates' physical properties. SRA correlated with BD, Mw, Mv, Por and CWC. SRL correlated with Mv and CWC.

**Table 4:** Mean morphological characteristics of roots growing on a different substrate.

Variants	Length (mm)	Diameter (mm)	Weight (mg)	SRA (m^2^ kg^−1^)	RTD (kg m^−3^)	SRL (m kg^−1^)
Soil	219.95 ± 65.14^a^	0.83 ± 0.12^a^	32.28 ± 11.18^a^	18.07 ± 1.43^b^	27.04 ± 3.80^a^	70.15 ± 11.69^a^
Deadwood	182.05 ± 91.08^b^	0.83 ± 0.11^a^	26.99 ± 14.53^b^	20.97 ± 5.51^a^	28.77 ± 8.98^a^	82.04 ± 24.14^a^

Mean ± standard deviation; seedlings growing on soil n = 30, seedlings growing on deadwood n = 30; root tissue density (RTD), specific root area (SRA) and specific root length (SRL).

^a,b^Statistically significant parameters; *p* < 0.05.

**Table 5:** Correlation between different root parameters and substrates properties.

	N	C	C/N	C/P	N/P	P	pH H_2_O	BG	DH	Ca	K	Mg	Na	BD	Mw	Mv	Por	Pa	CWC
Length	−0.14	−0.22	−0.13	−0.39*	−0.43*	0.31*	0.01	−0.06	−0.24	−0.33*	0.11	−0.33*	0.01	0.19	−0.22	−0.23	−0.21	−0.15	−0.29*
Diameter	0.21	0.05	−0.13	−0.02	0.01	0.12	−0.05	−0.09	−0.10	0.02	0.03	0.00	0.03	−0.06	0.01	−0.02	0.08	0.09	0.05
Weight	0.00	−0.11	−0.17	−0.36*	−0.41*	0.42*	0.01	0.00	−0.17	−0.28*	0.23	−0.26*	0.12	0.09	−0.12	−0.13	−0.09	−0.04	−0.19
SRA	−0.03	0.30*	0.40*	0.49*	0.48*	−0.30*	0.24	0.28*	0.36*	0.46*	−0.01	0.43*	0.15	−0.32*	0.30*	0.30*	0.30*	0.22	0.40*
RTD	0.06	0.04	−0.06	−0.13	−0.19	0.28*	0.02	0.10	0.04	−0.09	0.23	−0.05	0.16	−0.03	0.06	0.06	0.03	0.06	−0.03
SRL	−0.12	0.23	0.40*	0.42*	0.40*	−0.30*	0.23	0.28*	0.35*	0.38*	−0.01	0.36*	0.12	−0.25	0.25	0.27*	0.21	0.15	0.31*

*Correlation significant with *p* < 0.05*.*

**Figure 3:**
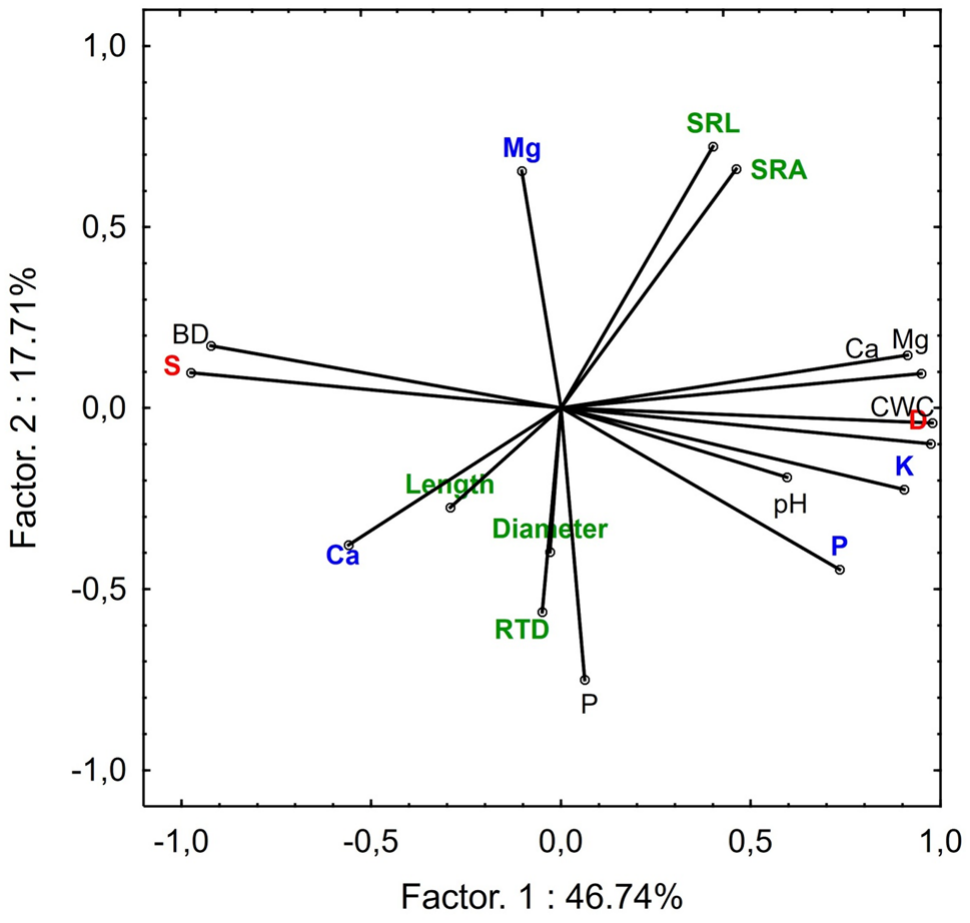
Diagram of PCA with projection of variables on a plane of the first and second factor for different substrates (*S *soil; *D* deadwood; blue colour—properties of needles; black colour—properties of substrate).

The PCA analysis confirmed the relationship between the substrate properties, the roots' morphology, and the seedlings' nutritional status (Fig. 3). Two main factors had a significant total impact (64.45%) on the variance of the variables. Factor 1 explained 56.74% of the variance of the examined properties. In contrast, factor 2 accounted for 17.71% of the variance (Fig. 3). Factor 1 is related to the properties of the substrates, while factor 2 is related to the morphological features of the roots. Deadwood properties noticeably differed from those of soil, with higher levels of P and K in needles associated with seedlings growing on deadwood. Additionally, deadwood has a higher Ca and Mg content and a higher pH and CWC. The length, diameter and RTD of roots strongly correlate with the needles' Ca and Mg content and the substrate's P content.

## Discussion

The substrate on which the silver fir seedlings grew exhibited distinct physical, chemical, and biochemical properties that influenced root morphology and nutritional status. Notably, heavily decomposed fir logs proved a favourable substrate for fir seedlings' growth. This was primarily due to their high content of exchangeable cations, including calcium, magnesium and potassium, as well as elevated levels of phosphorus and nitrogen. The increase in the content of nutrients in the decaying logs of different species was found in earlier studies^33^. The advanced decay stage of wood is characterised by a relatively efficient decomposition of lignocellulosic fibres, resulting in a sufficient amount of available forms of nitrogen within the wood. A strong release of basic cations from decomposing logs was reported in previous studies^1^. Consequently, seedlings growing on decaying wood displayed higher N, P, K, Mn, and Zn content than those growing on mineral soil^19^. The significantly higher K content in the needles of seedlings grown in deadwood indicates a more efficient K uptake in roots. Potassium plays a crucial ecological role in various processes, including photosynthetic activity, stomata-opening control, water movement, solute transport, control of osmotic pressure in the xylem, and the long-distance flow of sap from roots to shoots^34^. In decaying fir logs, significantly higher enzyme activity (dehydrogenases and β-glucosidase) was found, confirming higher microbiological activity than soil. Previous studies indicate the importance of the degree of decaying wood in shaping the number and diversity of bacteria, fungi and archaea^35–39^. The strong positive correlation between enzymatic activity and the content of basic cations suggests the involvement of microflora inhabiting deadwood in releasing these nutrients and indirectly affecting their availability to growing seedlings. These findings are consistent with previous studies^38,40,41^, in which it was shown that microorganisms and especially fungi catalyse transformations and decomposition processes of deadwood, thus contributing to the release of nutrients previously associated in wood. There are also reports that scarce nutrients during the decomposition of plant material can be actively transported through the mycelium from the surrounding soil to the decomposed substrate, thereby increasing the concentration of nutrients in the minimum, limiting the development of organisms^42–44^.

Furthermore, the results of this study indicate a trend in the differences between deadwood and soil in terms of microbial biomass carbon and nitrogen, although these differences were not statistically significant. The levels of MBC and MBN were lower in soils compared to deadwood, implying potential advantages for the growth of silver fir seedlings in deadwood. The quantity of soil microorganisms is closely related to both the quantity and quality of soil organic matter. A decrease in SOC leads to a decrease in soil microorganisms abundance^45,46^.

Decaying wood also exhibits more favourable physical properties compared to soil. The research revealed significantly higher overall porosity of fir logs than soil. Additionally, deadwood logs maintained higher humidity levels. Adequate water supply, as well as favourable air ratios, determine the appropriate growth of silver fir seedlings. Heavily decomposed wood boasts a higher water-holding capacity compared to mineral soils, and it's on par with peat^19^. The average moisture content of fallen logs varies during the vegetation period^15,47^, subject to microsite conditions. Shaded areas tend to retain higher moisture levels, while logs exposed to intense sunlight can experience drying, leading to fluctuating humidity levels. The influence of microclimatic conditions and topography on deadwood decomposition processes has been confirmed in earlier studies^48–50^. In conditions of stable moisture of decaying wood, fir seedlings found optimal growth conditions, resulting in characteristics similar to seedlings growing in a peat substrate^51^. It's important to consider both light and humidity conditions when assessing tree seedlings' growth and their nutrition status^52^. As the decomposition of deadwood progresses, its moisture content increases, which significantly affects the structure of microorganisms inhabiting the wood and processes occurring in it^53^.

Our research confirmed the differences in root morphology of a new generation of fir growing on deadwood and soil. Silver fir seedlings growing on the soil exhibited longer and more substantial root systems, suggesting the need to extend their roots to increase contact with the soil. This disparity in root characteristics may be related to the physical characteristics of the wood in the deeper parts of the logs. In the case of soil, the physical properties of the soil are relatively homogeneous over a dozen centimetres deep. The effect of bulk density and other soil physical characteristics on root morphology has also been shown in earlier studies^54,55^. In our study, specific root area (SRA) and specific root length (SRL) were the most responsive root traits to differences in growing substrates. While statistically significant differences were observed in SRA, SRL was higher (though not statistically significant) in seedlings growing on decaying wood. Specific root length is a commonly used parameter for assessing the effects of environmental changes on fine roots^56^. Fir seedlings growing on deadwood had higher SRA and SRL than the new generation of fir growing on soil. It can be assumed that higher SRA and SRL resulted in higher nutrient uptake efficiency per unit of root weight or length in seedlings growing on deadwood. Higher porosity was noted in the wood samples. Improvement of aeration results in better absorption of nutrients and, consequently, accelerates the growth and efficiency of plants^57^.

Our research confirmed that fir seedlings found equally favourable growth conditions in soil and decaying deadwood. Despite a wider C/N/P ratio and lower total biomass of the seedlings in deadwood compared to soil, seedlings are characterised by a similar nutritional status, which indicates no limitations in the uptake of nutrients, especially N and P from wood. There were no significant differences in the C/N, C/P and N/P ratios in the roots and needles of seedlings growing on the different substrates. There is a tendency to narrow the stoichiometry of the roots and needles of fir seedlings growing on wood. In previous studies, N/P stoichiometry has been used to assess nutrient deficits^58,59^, and an N/P ratio above 20 is associated with poorer biomass growth. In our study, the N/P of needles and roots of seedlings grown on soil and the needles of seedlings grown on wood and soil exceeded 20. These results indicate that both roots and needles responded to different substrates they grew in and should be evaluated simultaneously. Decaying wood can serve as a microhabitat with favourable physicochemical and biological properties for the growth of the young silver fir generation. Using highly decomposed deadwood as a substrate for seedlings' growth can enhance growth characteristics and nutritional status.

## Conclusions

The study highlights the substantial differences in the physical, chemical and, biochemical properties of the substrates (soil and deadwood) on which fir seedlings grow. These differences play a pivotal role in shaping the nutritional status of seedlings and their root morphology. Fir seedlings growing on decaying wood exhibited a notably higher content of nutrients. Furthermore, wood logs in advanced stages of decomposition feature properties that support the development of belowground biomass in seedlings. In soil, fir seedlings develop longer and more substantial root systems. However, when seedlings grow on deadwood, specific root area and specific root length are higher, indicating increased nutrient uptake efficiency. The stoichiometry of both roots and needles suggests no limitations in the uptake of nutrients by seedlings growing on deadwood. To enhance our understanding, further research should consider continuous monitoring of deadwood properties throughout the year, which would illustrate property variability, particularly in terms of moisture content. This factor may play a significant role in limiting the growth of new generations of trees.

## Data Availability

The datasets generated and analysed during the current study are not publicly available due to legal reasons but are available from the author upon reasonable request. Contact person-Ewa Błońska (ewa.blonska@urk.edu.pl).
